# Nosocomial transmission of *tet(x3)*, *bla*_NDM-1_ and *bla*_OXA-97_-carrying *Acinetobacter baumannii* conferring resistance to eravacycline and omadacycline, the Netherlands, March to August 2021

**DOI:** 10.2807/1560-7917.ES.2024.29.28.2400019

**Published:** 2024-07-11

**Authors:** Ditmer T Talsma, Rodrigo Monteiro, Rosario del Carmen Flores-Vallejo, Maarten Heuvelmans, Thuy-Nga Le, Antoni PA Hendrickx, Sigrid Rosema, Ianthe Maat, Jan Maarten van Dijl, Erik Bathoorn

**Affiliations:** 1 Department of Medical Microbiology, University of Groningen, University Medical Center Groningen, Groningen, The Netherlands; 2 Department of Medical Microbiology, Rivierenland Ziekenhuis, Tiel, The Netherlands; 3 Centre for Infectious Disease Control, National Institute for Public Health and the Environment (RIVM), Bilthoven, The Netherlands; 4 Radboud Center for Infectious Diseases, Department of Medical Microbiology, Radboud University Medical Center, Nijmegen, The Netherlands

**Keywords:** CRAb, outbreak, NDM, tet(x3), The Netherlands, healthcare-associated infections, bacterial infections, infection control, multidrug resistance, surveillance, molecular methods, typing

## Abstract

Carbapenem-resistant *Acinetobacter baumannii* (CRAb) is an important pathogen causing serious nosocomial infections. We describe an outbreak of CRAb in an intensive care unit in the Netherlands in 2021. During an outbreak of non-resistant *A. baumannii*, while infection control measures were in place, CRAb isolates carrying highly similar *bla*_NDM-1_ *-* and *tet(x3)-*encoding plasmids were isolated from three patients over a period of several months. The chromosomal and plasmid sequences of the CRAb and non-carbapenemase-carrying *A. baumannii* isolates cultured from patient materials were analysed using hybrid assemblies of short-read and long-read sequences. The CRAb isolates revealed that the CRAb outbreak consisted of two different strains, carrying similar plasmids. The plasmids contained multiple antibiotic resistance genes including the tetracycline resistance gene *tet(x3)*, and the *bla*_NDM-1_ and *bla*_OXA-97_ carbapenemase genes. We determined minimal inhibitory concentrations (MICs) for 13 antibiotics, including the newly registered tetracycline antibiotics eravacycline and omadacycline. The CRAb isolates showed high MICs for tetracycline antibiotics including eravacycline and omadacycline, except for minocycline which had a low MIC. In this study we show the value of sequencing multidrug-resistant *A. baumannii* for outbreak tracking and guiding outbreak mitigation measures.

**Key public health message**

**What did you want to address in this study and why?**

We wanted to uncover transmission routes of antimicrobial-resistant bacterial pathogens to improve infection prevention in the hospital environment. Since carbapenem-resistant *Acinetobacter baumannii* are rare in the Netherlands, we did a genetic analysis of the *A. baumannii* isolates that had acquired carbapenemase genes, in order to detect their genetic relationship and to infer their patient-to-patient transmission route.

**What have we learnt from this study?**

Carbapenemase-producing *A. baumannii* isolated from patients in the Netherlands can contain the *tet(x3)* gene, resulting in resistance to the tetracycline group of antibiotics. This gene has so far only been found in Asia. Since the respective patients had not travelled abroad, they probably acquired the multidrug-resistant *A. baumannii* in the hospital environment.

**What are the implications of your findings for public health?**

We have identified *A. baumannii* isolates that acquired unique genetic material. Some of the antibiotic resistance regions on this genetic material were never before detected in Europe. This study also shows the difficulty in controlling *A. baumannii* outbreaks once the hospital environment has been contaminated.

## Background

Carbapenem-resistant *Acinetobacter baumannii* (CRAb) is an important pathogen causing serious nosocomial infections with limited treatment options [1]. The World Health Organisation ranked CRAb with the highest score on their priority list to guide the development of new drugs [2]. *Acinetobacter baumannii* is a common cause of healthcare- or ventilator-associated pneumonia with an overall mortality rate of 43% [3]. The prevalence of CRAb is highly variable across Europe, with proportions varying from < 2.6% in northern European countries to 75.5% in southern European countries [4]. In the Netherlands, CRAb has rarely been detected in patients without a history of hospitalisation abroad.

Antibiotics of the tetracycline class are important last-line treatment options for CRAb infections (e.g. tigecycline, minocycline and the recently approved eravacycline and omadacycline) [5]. Tetracyclines inhibit bacterial protein synthesis by blocking the aminoacyl-tRNA binding site on the ribosome [6]. Acquired resistance to tetracyclines has been found in Gram-negative bacteria. One of the resistance mechanisms in Gram-negative bacteria is based on acquisition of the so-called *tet*(x) variants. *Tet(x)* genes code for monooxygenases that degrade a broad spectrum of tetracycline analogues [7]. Recent reports from China have described the occurrence of *tet(x)* genes on plasmids of Gram-negative bacteria, conferring high-level resistance to tigecycline [7,8]. In carbapenem-resistant *Acinetobacter* species, the detection of *tet(x)* genes on plasmids has been reported, and the authors recommended surveillance of the dissemination of *tet(x)* carrying plasmids [9]. To date, to the best of our knowledge, there have not been any reports of *tet(x)* detection in clinical specimens from Europe.

In the present report, we describe an outbreak involving closely related CRAb in the Netherlands. We have characterised the large multidrug resistance (MDR) plasmids present in both outbreak clones, carrying *tet(x3)*, the two carbapenemase genes *bla*_NDM-1_ and *bla*_OXA-97_, and numerous other resistance genes. Further, we investigated the impact of *tet(x3*) on the susceptibility to antibiotics of the tetracycline class, including eravacycline and omadacycline.

## Outbreak detection

**Figure 1:**
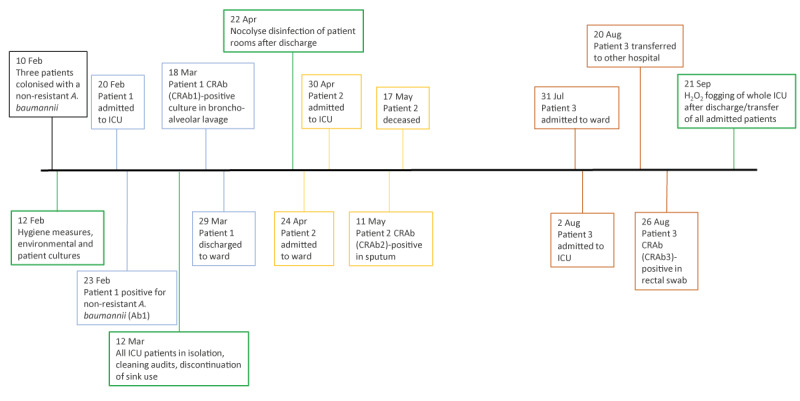
Timeline of cultures and preventive measures, nosocomial outbreak of carbapenem-resistant *Acinetobacter baumannii*, the Netherlands, March–August 2021 (n = 3)

In early February 2021, several patients treated for COVID-19 in the intensive care unit (ICU) of a Dutch hospital tested positive for non-resistant *A. baumannii*. In mid-March, one of these patients was found to be colonised with CRAb. This index patient (Patient 1) had been colonised with a non-resistant *A. baumannii* isolate (denoted Ab1) from Day 3 after their admission, and this isolate was persistently cultured from respiratory material of the patient in the subsequent weeks. Four weeks after the admission, the first CRAb isolate (denoted CRAb1) was cultured from bronchoalveolar lavage fluid of the index patient (Figure 1). Since the index patient had no recent travel history, it was suggested that they had contracted the CRAb on the ICU. The index patient could be transferred to the regular ward 1 week after culturing of the CRAb.

Sequential infection prevention measures (green) and culture and discharge/transfer data of patient 1 (blue), patient 2 (yellow) and patient 3 (red).

Despite infection prevention measures, another patient (Patient 2) admitted for infection with severe acute respiratory syndrome coronavirus 2 (SARS-CoV-2), presented a CRAb-positive sputum sample in mid-May (solely culture-positive in sputum) (Figure 1). The respective isolate was denoted CRAb2. This CRAb2-carrying patient had been admitted to the ICU 1.5 months after discharge of the index patient and had tested negative for *A. baumannii* in multiple rectal screening cultures before testing positive for CRAb. This patient passed away 5 days after testing positive for CRAb due to respiratory failure. It remained unclear to what extent this outcome was affected by the CRAb carriage, although treatment consisting of ciprofloxacin 400 mg three times daily combined with nebulised colistin was started when the CRAb was isolated from the patient’s respiratory tract. 

In the following 2.5 months, no further wild-type *A. baumannii* or CRAb isolates were cultured from patient material collected at the ICU where the outbreak had occurred. However, 2.5 months after the CRAb2-positive patient had passed away, a third patient (Patient 3), who had been staying at this same ICU for 3 weeks before transfer to another hospital, tested positive for CRAb in a rectal swab 6 days after transfer (Figure 1). This patient’s isolate was denoted CRAb3. Despite extensive culturing, solely rectal colonisation was detectable for Patient 3. Patient 3 passed away in October due to respiratory failure unrelated to the colonisation with the CRAb.

## Methods

### Setting and definitions

In this report, we describe an outbreak of CRAb in a five-bed ICU within a 300-bed regional hospital in the period between March and August 2021. Standard surveillance for multidrug-resistant organisms on this ICU consisted of nose, throat and rectal swabs once a month, which were tested for meticillin-resistant *Staphylococcus aureus*, vancomycin-resistant *Enterococcus faecium* and Gram-negative bacteria producing extended spectrum β-lactamases or carbapenemases. No CRAb isolate had been isolated from patients in this hospital before this outbreak. A total of three CRAb isolates were collected from three different patients, all admitted to the ICU. All clinical isolates in this study were tested for antibiotic susceptibility using the Becton, Dickinson and Company Phoenix automated identification and susceptibility testing system. Carbapenem resistance was confirmed by meropenem gradient strip test (E-test, bioMérieux, Amersfoort, the Netherlands). Carbapenemase gene carriage was determined by whole genome sequencing (WGS) as outlined below.

### Rectal and environmental culture screening protocol

Rectal swabs were incubated in brain heart infusion broth (Thermo Fisher Scientific, Waltham, United States (US)) containing 16 mg/L amoxicillin and incubated for 24 h at 35 °C, whereafter 100 µL of broth was used to inoculate Brilliance CRE agar (Thermo Fisher Scientific), CHROMID ESBL agar and CHROMID Oxa-48 agar (bioMérieux). The plates were then incubated for 48 h at 35 °C. Environmental samples were collected when active transmission of *A. baumannii* was suspected, and plated on 5% sheep blood agar (BA) and McConkey agar for 48 h at 35 °C.

### Additional testing of outbreak strains

For the purpose of this study, we performed additional susceptibility testing and WGS on three isolates involved in the outbreak. This concerned the initial wildtype antibiotic-sensitive *A. baumannii* isolate of the index patient (Ab1), the CRAb isolate of the index patient (CRAb1), and a CRAb isolate of the first consecutive positive patient in the outbreak (CRAb2). The isolate of the third CRAb positive patient in the outbreak was named CRAb3. This CRAb3 isolate was isogenic to the CRAb2 isolate, and additional susceptibility testing was therefore not performed.

### Antibiotic susceptibility testing

Antibiotic susceptibility testing (AST) was performed using VITEK-2 (card N344), gradient strip tests, and the broth microdilution (BMD) method. The MICs for meropenem, imipenem, ampicillin/sulbactam and tigecycline were determined using the gradient strip method (bioMérieux) protocol. We made 0.5 McFarland suspensions in 0.9% (w/v) saline from 1-day-old cultures on BA plates. The isolates were inoculated on Mueller Hinton (MH) agar from Mediaproducts, Groningen, the Netherlands. All the plates were incubated at 35 °C for 16–20 h after which the minimum inhibitory concentration (MIC) was determined. The MIC was defined as 100% growth inhibition.

Susceptibility testing for minocycline, eravacycline and omadacycline was performed using the BMD method. For minocycline, MICs were determined by BMD in cation-adjusted MH broth (TREK diagnostic systems/Thermo Fisher), according to the Clinical and Laboratory Standards Institute (CLSI) guidelines [36]. Minocycline MIC was read with 80% inhibition of growth (compared against the growth control), when trailing growth occurred. Eravacycline and omadacycline BMD testing was performed according to ISO 20776–1:2019 using the broth culture method to prepare the inoculum. We interpreted MICs according to the European Committee on Antimicrobial Susceptibility Testing (EUCAST) guidelines with tetracycline as a control [10,11]. The *A. baumannii* ATCC 17978 and *Escherichia coli* ATCC 25922 strains were included as reference and quality control. All the concentrations were tested in triplicates in two independent experiments (n = 6). As negative controls, we included bacteria treated only with MH broth and bacteria treated with the final concentration of dimethyl sulfoxide in the assay (vehicle ≤ 0.31% v/v_final_). The MIC was determined visually by naked-eye inspection and defined as the lowest concentration of antibiotic that completely inhibited bacterial growth (no haze growth, pinpoint growth or turbidity was seen) upon incubation at 35 °C for 24 h. With the aim of assisting the determination of the MIC with a complementary spectrophotometric method, end-point absorbance measurements (Abs_λ = 600 nm_) were done using a Biotek Epoch-2 reader. The results were graphed using GraphPad Prism V.8.0.1 according to the following equation:

Bacterial growth (%) = (mean OD_600_ value of antibiotic-treated bacteria _(at t = 24 h)_ − mean OD_600_ value of antibiotic-treated bacteria _(at t = 0 h)_) / (mean OD_600_ value of vehicle-treated bacteria _(after t = 24 h)_ − mean OD_600_ value of vehicle-treated bacteria _(at t = 0 h)_) × 100. 

In the absence of published EUCAST breakpoints for tetracycline-related antibiotics against *Acinetobacter* species, the interpretation of the MIC was done based on the CLSI guidelines [37].

### Whole genome sequencing

#### Illumina short-read sequencing

A total DNA extraction for WGS was performed directly from colonies of the different *A. baumannii* isolates using an Ultraclean Microbial DNA Isolation Kit (MO BIO Laboratories, Carlsbad, US). We determined DNA concentrations using a Qubit 2.0 fluorometer and the dsDNA HS and/or BR assay kit (Life Technologies, Carlsbad, US) and prepared DNA libraries using the Nextera XT v2 kit (Illumina, San Diego, US). Short-read sequencing was performed with an Illumina MiSeq System, generating paired-end reads of 250 bp. De novo assembly of paired-end reads was performed using CLC Genomics Workbench v20.0.4 (QIAGEN, Hilden, Germany) after quality trimming (Qs ≥ 20), establishing a word size of 29.

#### Nanopore long-read sequencing

High-molecular weight DNA was isolated using an in-house developed protocol [12], and the Oxford Nanopore protocol SQK-LSK109 was followed (Oxford Nanopore Technologies, Oxford, United Kingdom). The final library was loaded onto a MinION flow cell (MIN-106 R9.4.1) and a 48 h sequence run was started without live base calling. Once the sequencing run was completed, we performed base-calling and de-multiplexing using Albacore v2.3.1 and extracted a single FASTA file per isolate from the FAST5 files using Poretools v0.5.1 [13]. At both sides, 50 bp were trimmed, and only reads larger than 5,000 bp were used in further analyses.

#### Hybrid assembly of Illumina and Nanopore DNA sequences

The Nanopore and Illumina sequencing data were used for hybrid genome assembly using Unicycler v0.4.4 [14]. The resulting contig files were annotated using Prokka v1.14.6 and loaded into BioNumerics version 7.6.3 (Applied Maths, Sint-Martens-Latem, Belgium) for further comparative analyses [15]. Plasmids with ≥ 95% sequence identity were regarded as highly similar. Lastly, we analysed the plasmids by using the basic local alignment search tool (BLAST) from the National Center for Biotechnology Information (NCBI).

### Sequence data analysis for antimicrobial resistance determinants and plasmids

The antibiotic resistance gene profiles and plasmid replicons of all of sequenced *A. baumannii* isolates were assessed using the AMRFinder version 3.8.4, IslandViewer4 and PlasmidFinder version 2.0.2 databases available from the Center for Genomic Epidemiology [16,17]. For AMRFinder, we used a 90% identity and a minimum length of 60% threshold, and an identity of 95% for PlasmidFinder. The resulting sequence-derived data, such as resistance genes, replicons and whole-genome multiple-locus sequence type (wgMLST) profiles were imported into BioNumerics for analysis. For comparative wgMLST analyses, we used the short-read sequencing data of the *A. baumannii* isolates and built a minimum spanning tree (MST) using BioNumerics as described previously [12]. The categorical coefficient was used to calculate the MST, and the MST was based on an in-house *A. baumannii* wgMLST scheme made in SeqSphere software version 6.0.2 (Ridom GmbH, Münster, Germany). The in-house *A. baumannii* wgMLST scheme was based on 3,473 genes, including 2,390 core genome genes and 1,083 accessory genome genes, using the genome of the *A. baumannii* strain ACICU (GenBank accession number NC_010611.1, September 2021) as a reference.

## Results

### Antimicrobial susceptibility testing of *Acinetobacter baumannii* outbreak isolates

The results of the AST of the Ab1, CRAb1 and CRAB2 isolates are presented in the Table. The WGS showed that the CRAb3 isolate was genetically nearly identical to the CRAb2 isolate, and it was therefore not subjected to extensive AST. In particular, the Ab1 isolate showed MICs below the CLSI breakpoints to all tested antibiotics with the exception of an intermediate resistance to tetracycline. In contrast, the CRAb1 and CRAb2 isolates showed MICs above the CLSI breakpoint for ampicillin/sulbactam and carbapenems. Moreover, the MICs were high for most of the tetracycline antibiotics, including tigecycline, as well as eravacycline and omadacycline; the detailed results are appended in Supplementary Figure S1. The MICs for minocycline were low, both in the Ab1 strain and the CRAb1 and CRAb2 strains. For comparison, we tested the MICs of the reference strains *A. baumannii* ATCC 17978 and *E. coli* ATCC 25922 in parallel; they were identical to the reference MICs provided by EUCAST and CLSI (Table).

**Table:** Antibiotic susceptibility and resistome of *Acinetobacter baumannii* isolates, hospital outbreak, the Netherlands, March–August 2021 (n = 3)

Isolate	Previous ICU stay (days)	Isolation date	Susceptibility to CIP^a^/STX^a^/GEN^a^/TOB^a^	MIC IMI^c^	MIC MERO^c^	MIC AMP/SUL^c^	MIC TET^b^	MIC MINO^c^	MIC TIGE^c^	MIC ERA^b^	MIC OMA^b^	*bla-* genes^d^	Non *bla-resistance genes^d^*
Ab1	3	23 Feb 2021	I/S/S/S	0.19	0.38	1.0	8	0.12	0.38	0.125	0.5	*bla*_ADC-79_, *bla*_OXA-100_	-
CRAb1	26	18 Mar 2021	I/R/S/S	> 32	> 32	48	128	0.12	> 4.0	4	16	*bla*_ADC-79_, *bla*_OXA-100_, *bla*_NDM-1_, *bla*_OXA-97_	*tet(x3), sul2, ant(2”)-la, aph(3”)-lb, aph(3’)-la, aph(6)-ld, ant(3”)-lla, mph(E), msr(E)*
CRAb2	12	11 May 2021	I/R/R/S	> 32	> 32	64	64	0.25	> 4.0	2	8	*bla*_ADC-79_, *bla*_OXA-100_, *bla*_NDM-1_, *bla_OXA-97_*	*tet(x3), sul2, abaF, ant(2”)-la, aph(3”)-lb, aph(3’)-la, aph(6)-ld, ant(3”)-lla, mph(E), msr(E)*
*Escherichia coli* ATCC 25922	NA	NA	NT	0.125	0.023	3.0	1.0	0.50	0.094	0.125	0.5	NT	NT
*Acinetobacter baumannii* ATCC 17978	NA	NA	NT	0.19	0.38	1.0	2.0	0.12	0.25	0.063	0.125	NT	NT

AMP/SUL: ampicillin/sulbactam; CIP: ciprofloxacin; ERA: eravacycline; GEN: gentamicin; ICU: intensive care unit; IMI: imipenem; MERO: meropenem; MIC: minimum inhibitory concentration; MINO: minocycline; NA: not applicable; NT: not tested; OMA: omadacycline; STX: co-trimoxazol; TET: tetracycline; TIGE: tigecycline; TOB: tobramycin.

^a^ MIC determined by VITEK-2.

^b^ MIC determined by broth microdilution.

^c^ MIC determined using the gradient strip method.

^d^ Antimicrobial resistance genes as identified with AMRFinder.

The MICs are presented in mg/L.

### Genomic analysis of the outbreak *Acinetobacter baumannii* isolates

The WGS showed that the Ab1 and CRAb1 isolates belonged to MLST sequence type (ST)1060, while the CRAb2 and CRAb3 isolates belonged to MLST ST823. The Ab1 isolate and the CRAb1 isolate, which were isolated from the same patient, were the same strain, showing a difference of only one allele. The analysis also revealed that the CRAb2 and CRAb3 isolates from Patients 2 and 3, respectively, were the same strain with only one allele difference. However, CRAb2 and CRAb3 belonged to a different wgMLST cluster than the Ab1 and CRAb1 isolate, with 238 allelic differences between the clusters.

Analysis of the hybrid genome assemblies with AMRFinder revealed the presence of the intrinsic chromosomally encoded resistance genes of *A. baumannii* in the three sequenced isolates Ab1, CRAb1 and CRAb2. These included, among others, the *bla*_OXA-100_ and *bla*_ADC-79_ genes (Table). The isolate CRAb2 carried the fosfomycin resistance gene *abaF* chromosomally, whereas the isolate CRAb1 lacked this gene.

### Analysis of multidrug resistance plasmids

The isolate CRAb1 was found to harbour a resistance gene-carrying plasmid (pCRAb1) highly similar but not identical to the ones in the isolates CRAb2 (pCRAb2) and CRAb3 (pCRAb3). Plasmids pCRAb2 and pCRAb3 were identical. The length of pCRAb1 was 262 kbp and 265 kbp for pCRAb2, and pCRAb2 displayed 38 SNPs and some insertions and deletions in intergenic regions compared with pCRAb1 which are listed in Supplementary Table S1. In addition, pCRAb2 showed extra IS1202 elements, resulting in the > 3,000 bp larger plasmid sequence. These plasmids had not been published before in GenBank, and we additionally provide in Supplementary Figure S2 the phylogenetic tree of related *Acinetobacter* plasmids. 

**Figure 2:**
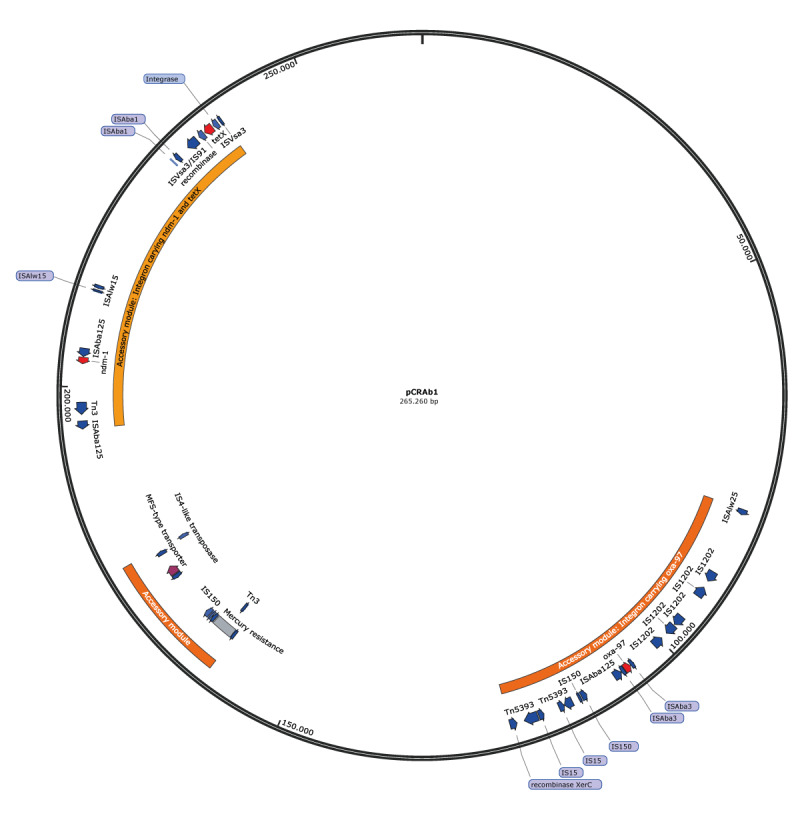
pCRAb1 plasmid map, *Acinetobacter baumannii* isolate, hospital outbreak, the Netherlands, March–August 2021

Since all plasmids were highly similar, we here present a more detailed analysis of only pCRAb1 (Figure 2). It was a conjugative plasmid that contains several resistance genes located on three accessory modules, including the beta-lactam resistance genes *bla*_NDM-1_, *bla*_OXA-97_, the tetracycline resistance gene *tet(x3)*, the sulphonamide resistance gene *sul2*, the aminoglycoside resistance genes *ant(2”)-la*,* aph(3”)-lb*,* aph(3’)-la*,* aph(6)-ld*, and the macrolide resistance genes *mph(E)* and *msr(E).* In addition, the plasmid carried several virulence-related genes, including genes encoding major facilitator superfamily (MFS) transporters, iron uptake proteins, lipoproteins and secretion systems [18-21]. Genes responsible for horizontal plasmid mobility, such as *traX*, *dotL*, *dotM*, *dotO*, and a gene encoding a Trbl-like protein [22-24] were encoded by this plasmid. For more information, we refer to Supplementary Table S2, which lists the complete annotation of the pCRAb1. Lastly, the plasmid carried three genomic islands (GI), each harbouring antibiotic resistance and virulence-related genes.

Red: antibiotic resistance genes; dark blue: virulence-related genes. Other genes are not shown for simplification. Plasmid map generated by SnapGene.

**Figure 3:**
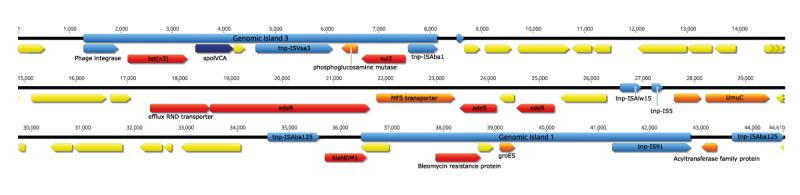
Coding sequences of the integron containing genomic island 1 and genomic island 3, *Acinetobacter baumannii* isolate, hospital outbreak, the Netherlands, March–August 2021

Of note, GI1 and GI3 were both located on an integron (Figure 3), which encodes the two relevant antibiotic resistance genes *bla*_NDM-1_ and *tet(x3)* as well as other genes related to efflux pumps, MFS transporters, DNA polymerase V, GroES, and mobility elements such as integrase and transposases [25]. In Supplementary Figure S3 we append an additional BLASTn comparison of this integron with publicly available sequences in NCBI, revealing that GI sequences highly identical to GI1 and GI3 have been reported previously, but never together on a single integron. The *tet(x3)*-carrying part of the integron (GI3) was highly similar (99% identity) to GI regions from different *Acinetobacter* species such as *A. indicus* and *A. portensis,* which are both environmental strains from China. The GI3 was 6,874 bp long and encoded a phage integrase, ISAba1 and IS91-like transposons, as well as a site-specific recombinase SpoIVCA, which may increase the mobility of this region and potentially be involved in the resistance transfer.

Blue: mobile elements-related genes; red: antibiotic resistance genes *tet(x3), bla*_NDM-1_ and *sul2*; yellow: hypothetical proteins, not labelled. Integron map generated by Geneious Prime 2023.2.1.

### Outbreak control measures

Before the detection of CRAb, outbreak prevention measures were already in place in the ICU because of an outbreak of non-resistant *A. baumannii* (including isolate Ab1) involving multiple patients. These measures included intensified discharge cleaning of nursing rooms, weekly patient screening by rectal culture on all patients admitted to the ICU, and environmental screening of medical devices used for multiple patients, tray tables, beds, and sinks. Despite these measures, *A. baumannii* was persistently cultured from patients and their environment. Moreover, all three CRAb-positive patients had been admitted to the ICU because of a SARS-CoV-2 infection and were therefore nursed in isolation or by cohort nursing with a negative pressure regime. Medical staff was wearing long-sleeve gowns, gloves, glasses and FFP2 masks. Upon culturing of an *A. baumannii *or CRAb isolate, the respective patients were placed in a single room with negative pressure regime. Medical staff was wearing long-sleeve gowns, gloves, surgical masks (or FFP2 masks in case the SARS-CoV-2 infection remained), glasses and hairnets. Patients testing positive for CRAb in clinical samples (e.g. sputum) were tested for confirmation by rectal swab and re-testing of the positive body site. 

After identification of the CRAb-positive index patient, infection prevention measures were further intensified: all patients in the ICU were nursed in isolation with daily cleaning of rooms and disposal of all unused disposable medical equipment upon discharge of patients, discontinued usage of sinks in patient rooms, special hand hygiene training for nurses and medical staff, and implementation of adenosine triphosphate measurements to aid cleaning of the patient area. Because non-resistant *A. baumannii* continued to be identified in environmental cultures, nocolysis of nursing rooms was performed after patient discharge. This stopped the transmission of non-resistant *A. baumannii,* and also environmental cultures remained negative after the start of nocolysis. Nonetheless, after the start of nocolysis, two more CRAb-positive patients were identified (CRAb2 and CRAb3). Because all possible measures had already been taken before identification of the second CRAb-positive patient, no additional measures were taken. However, upon identification of the CRAb3 isolate, an admission stop was instigated, and all patients on the respective ICU were either discharged or transferred to other hospitals in the following weeks. Once all patients had been discharged, the entire ICU was disinfected and cleaned twice, followed by hydrogen peroxide vapour decontamination. Hereafter, no more CRAb-positive patients were identified.

## Discussion

The present study describes an unusual outbreak of CRAb in a Dutch ICU, which was caused by two distinct clones of *A. baumannii* that both contained plasmid-borne carbapenemase *bla*_OXA-97_, *bla*_NDM-1_ and tetracycline resistance-conferring *tet(x3)* genes. The fact that the present CRAb outbreak involved two separate clusters representing distinct MLST types of *A. baumannii* is remarkable because the isolation of a CRAb from patient-derived materials is still a very rare event in Dutch hospitals. In fact, CRAb had never before been isolated from patients in the hospital in which the outbreak occurred. So far, the isolation of CRAb has always been associated with recent treatment of the patients in hospitals outside the Netherlands. Unexpectedly, however, the CRAb-positive patients described in our present study had not visited foreign hospitals in the recent past.

Since no CRAb was identified by environmental sampling, the precise mechanism of transmission remains elusive. In fact, we can also only hypothesise how the described outbreak of CRAb was caused by two different MLST types carrying similar plasmids. The first two patients involved in the outbreak had tested negative for *A. baumannii* in screening cultures before being colonised by CRAb, suggesting that they had acquired CRAb in the ICU. This also seems the most likely scenario for the detection of the genetically identical CRAb2 and CRAb3 isolates in Patients 2 and 3, where CRAb2 was most likely transmitted to Patient 3. The hypothesis that transmission had occurred in the ICU is supported by the fact that vigorous cleaning of the whole ICU, including the use of hydrogen peroxide vapour, effectively stopped the outbreak. Two plausible explanations for an outbreak of CRAb consisting of two distinct MLST types are that CRAb2 evolved from CRAb1 via missing links between the two isolates, or that they shared a common ancestor that had persisted in a local reservoir for a longer time period, combined with a transfer of the antibiotic resistance-carrying plasmid between CRAb1 and CRAb2/3. Unfortunately, we can only speculate on the exact mechanism of transmission and the relation between the two MLST types in the outbreak, since no CRAb isolates were recovered from environmental samples.

It is interesting to note that the isogenic Ab1 and CRAb1 isolates were obtained from the index Patient 1. This, as discussed above, indicates an introduction of the *bla*_NDM-1_ and *bla*_OXA-97-_carrying plasmid in close vicinity of Patient 1, which would be supported by the fact that none of the environmental cultures tested positive for CRAb. Also remarkable is the size of the plasmids (> 260 kb), which is due to the acquisition of an extensive accessory genome mediated by insertion sequences and phage integrases [26]. Only a limited number of plasmid lineages are known for *Acinetobacter*, and only some of these lineages include plasmids with multiple antibiotic resistance genes [27]. Such MDR plasmids are often large conjugative plasmids of more than 100 kb [28]. Analysis of the genomic islands and the integrase carrying the *bla*_NDM-1_ and *tet(x3)* in the plasmids of our outbreak revealed that the *tet(x3)-*carrying part had been a recent addition to the integrase. The CRAb2 and CRAb3 isolates, belonging to a different MLST type, carried a plasmid that was 98% identical to the plasmid carried by the CRAb1 isolate, which suggests a common recent ancestry.

The *tet(x3)* gene found on the plasmids conveys resistance to a broad range of antibiotics in the tetracycline group. Antibiotics from the tetracycline group are often regarded as a last line treatment for CRAb infections. Tetracycline resistance mediated by *tet(x)* genes was previously described upon the introduction of tigecycline [29,30] and it has been reported for clinical *A. baumannii* isolates in China [31]. Chen et al. also described the phenotypical resistance patterns for tetracyclines in *tet(x)*-carrying *A. baumannii*, including resistance to tetracycline and tigecycline, as well as high MICs for the new-generation tetracyclines omadacycline (8–16 mg/L) and eravacycline (2–8 mg/L) [31].

Interestingly, the CRAb isolates described in our present study were susceptible to minocycline. To date, minocycline MICs in *tet(x3)-*carrying *Acinetobacter* have not been described. However, the reported minocycline MIC for an *A. baumannii* isolate carrying *tet(x5)* was 2–8 mg/L, and also in *tet(x3)*- or *tet(x4)*-carrying *E. coli* isolates, the MICs for minocycline were increased compared with a *tet(x)*-naïve strain [8,32]. These observations suggest that minocycline may be a treatment option for infections caused by some, but certainly not all, *tet(x)*-carrying *A. baumannii*. This makes a precise MIC determination relevant, since it was recently demonstrated that minocycline could be a cornerstone in the treatment of multi- and extremely drug-resistant *A. baumannii* infections due to its synergy with sulbactam [33]. Dimitriadis et al. showed that the gradient strip method is less reliable than automated susceptibility testing for minocycline [34]. Moreover, the optimal dosage of minocycline for *A. baumannii* infections is a topic of debate, as recent pharmacokinetic and pharmacodynamic data of minocycline administration suggest that the current breakpoints might be too high [35]. The discussion on the optimal dosing and AST method for minocycline in *A. baumannii* might be even more relevant after to the introduction of a *tet(x3)*-carrying *A. baumannii* in Europe described in this study.

## Conclusion

We here report the occurrence of CRAb in the Netherlands with an extended resistance pattern to the last-line tetracycline antibiotics and ampicillin/sulbactam in hospitalised patients with no clinical history abroad. The transmissions were rapidly confirmed by collaborative typing efforts at an academic institution and the National Institute for Public Health and the Environment of the Netherlands. The immediate infection control interventions most probably prevented a large-scale outbreak.

## Supplementary Data

852000 Supplement
