# Supplementary material for: Nosocomial transmission of tet(x3), bla NDM-1 and bla OXA-97-carrying Acinetobacter baumannii conferring resistance to eravacycline and omadacycline, the Netherlands, March to August 2021
Source: Euro Surveill. 2024 Jul 11;29(28):2400019. doi: 10.2807/1560-7917.ES.2024.29.28.2400019 (PMC11241855; doi:10.2807/1560-7917.ES.2024.29.28.2400019)
Supplement: Supplement [file 24-00019_TALSMA_Supplement.pdf]

Supplemental material

This supplementary material is hosted by *Eurosurveillance* as supporting information alongside the article Nosocomial transmission of *tet(x3)*, bla_{NDM-1}, and bla_{OXA-97}-carrying *Acinetobacter baumannii* conferring resistance to eravacycline and omadacycline, the Netherlands, March-August 2021, on behalf of the authors, who remain responsible for the accuracy and appropriateness of the content. The same standards for ethics, copyright, attributions and permissions as for the article apply. Supplements are not edited by *Eurosurveillance* and the journal is not responsible for the maintenance of any links or email addresses provided therein.

Supplemental figure 1. Inhibitory effect of tetracycline class antibiotics against *A. baumannii* clinical isolates and reference strains: (a) Tetracycline, (b) Eravacycline, and (c) Omadacycline.

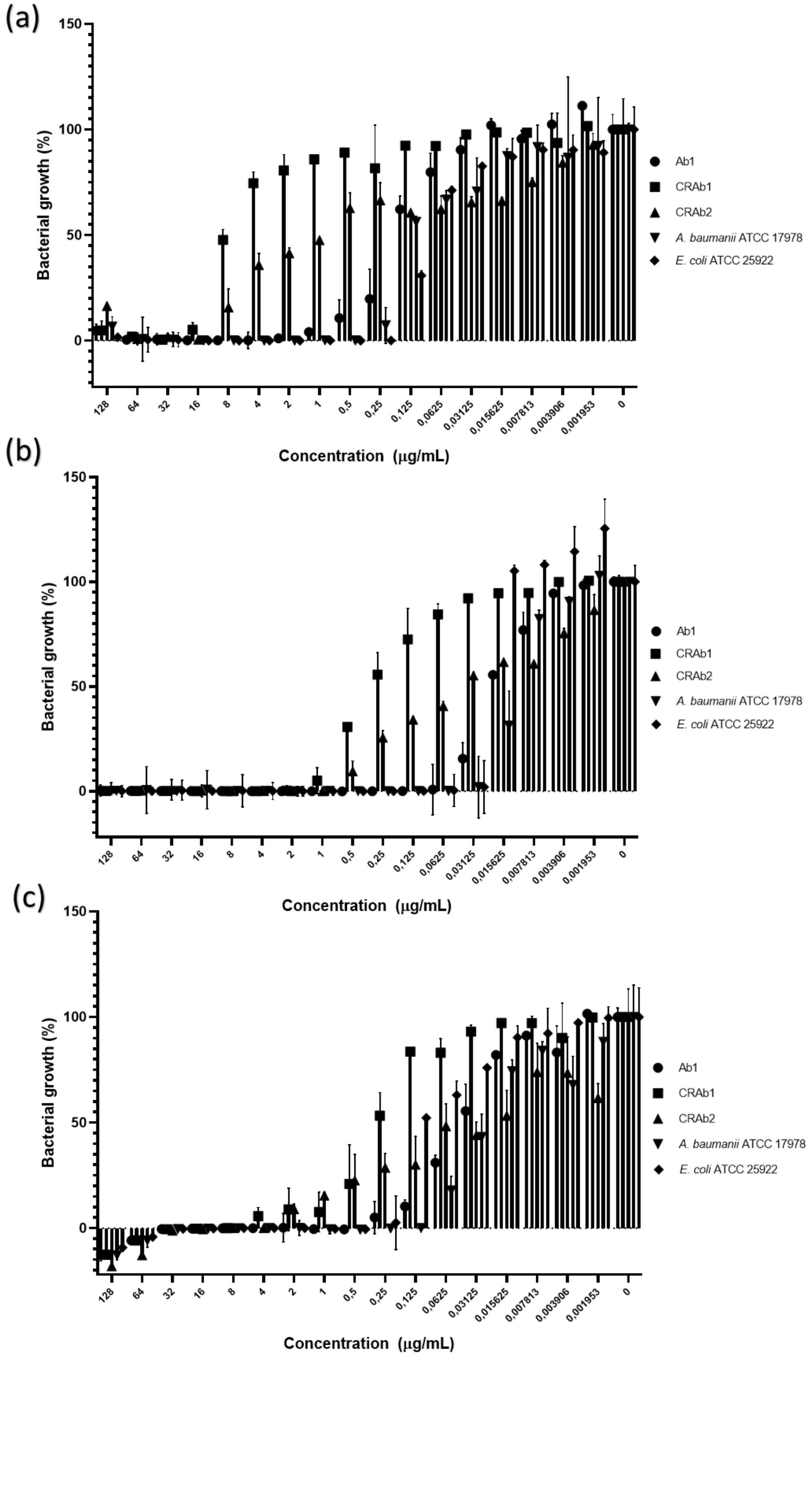

Supplemental figure 2. Phylogenetic tree of *A. baumannii* plasmids based on sequences submitted to Genbank, showing that the plasmid of the CRAb1 and CRAb2 isolates (highlighted in yellow) is unique (analysis performed in September 2021).

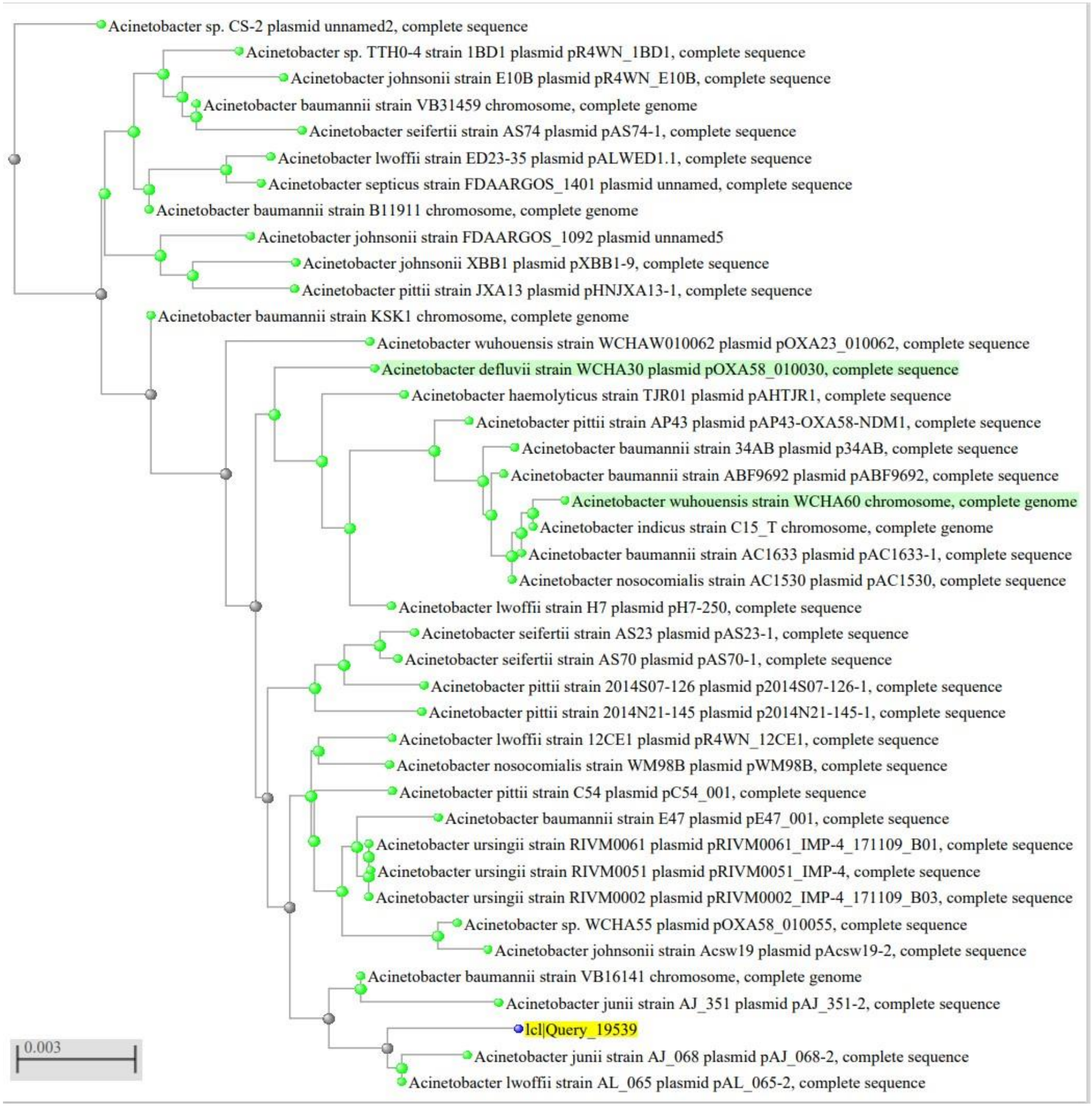

Supplemental figure 3. BLASTn of the *NMD-1* and *tet(x)3* carrying integron. Both the *NMD-1* and *tet(x)3* genes located within genomic islands 3 and 1, respectively, have been described before, but the plasmids identified in the present outbreak are unique in combining these resistance genes in one integron.

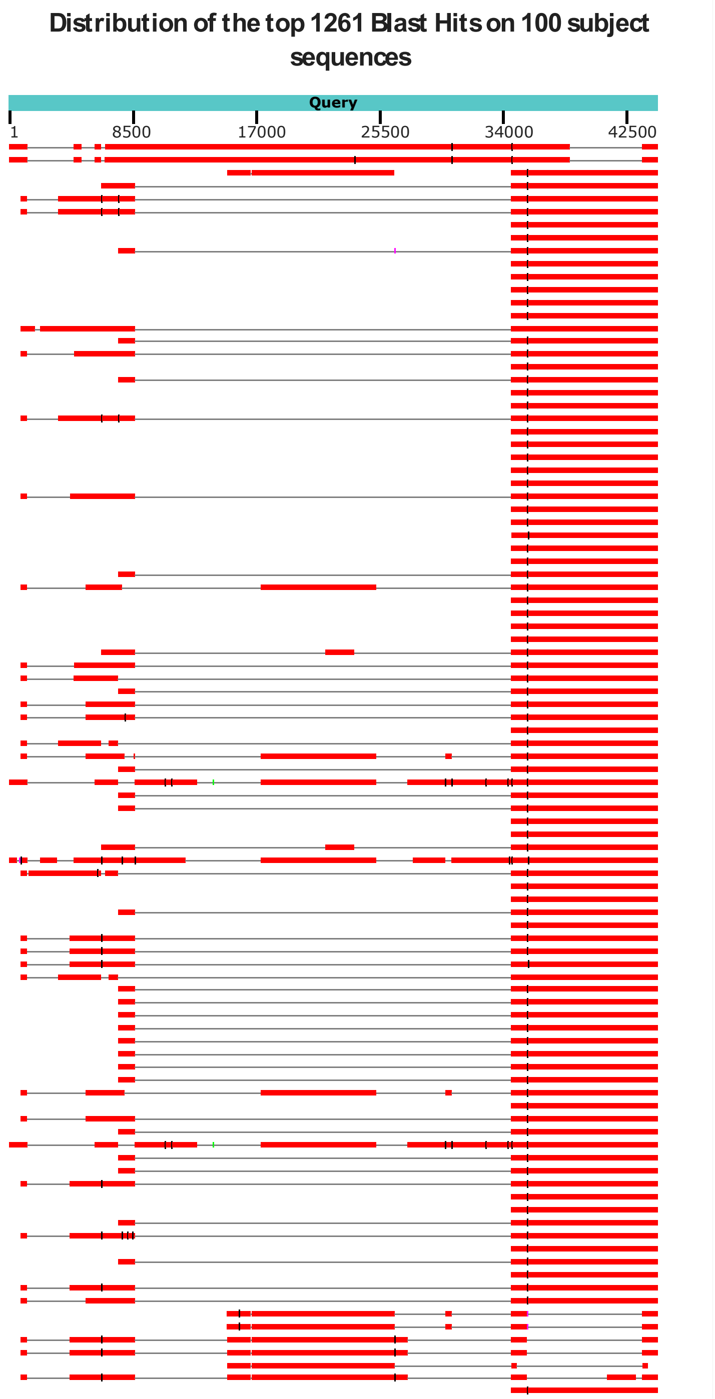

Supplemental table 1. SNP analysis of the plasmid sequence of CRAb1 compared to the plasmid sequence of CRAb2.

**SNP pattern**	Sequence	Positions		AA change	Codon	Gene
TC	CRAb2	7994	7994	No	CTT to CTC	ISAba125 family transposase
TC	CRAb2	8084	8084	No	ACT to ACC	ISAba125 family transposase
TC	CRAb2	8087	8087	No	TTT to TTC	ISAba125 family transposase
AC	CRAb2	8273	8273	No	CCA to CCC	ISAba125 family transposase
GT	CRAb2	14389	14389	S to Y	GGA to GTA	phosphohydrolase
GA	CRAb2	43225	43225	No	Intergenic region	
AT	CRAb2	53111	53111	K to STOP	AAG to TAG	DNA methyltransferase
GT	CRAb2	55868	55868	G to C	GGT to TGT	restriction endonuclease subunit R
AT	CRAb2	63505	63505	No (L)	AAG to TAG	Hypothetical protein
GA	CRAb2	78476	78476	No (K)	AAG to AAA	Hypothetical protein
GT	CRAb2	86290	86290	S to L	CGA to CTA	Transposase
GT	CRAb2	89003	89003	R to L	CGA to CTA	IS26 transposase
TC	CRAb2	96474	96474	No (L)	CTT to CTC	tnp-IS30
TC	CRAb2	96564	96564	No (T)	ACT to ACC	tnp-IS30
TC	CRAb2	96567	96567	No(F)	TTT to TTC	tnp-IS30
AC	CRAb2	96753	96753	No (P)	CCA to CCC	tnp-IS30
GA	CRAb2	96789	96789	No (L)	CTG to CTA	tnp-IS30
AG	CRAb2	99488	99488	No	Intergenic region	
TG	CRAb2	116693	116693	F to Q	TTT to TTG	LysR family transcriptional regulator
CG	CRAb2	143183	143183	No	Intergenic region	
TA	CRAb2	168599	168599	No (A)	GCT to GCA	LPD1 domain-containing protein
GA	CRAb2	173427	173427	No	Intergenic region	
TC	CRAb2	196031	196031	H to R	GTG to GCG	Hypothetical protein
GA	CRAb2	197758	197758	No (Y)	GTA to ATA	Hypothetical protein
CA	CRAb2	200511	200511	No	Intergenic region	
GA	CRAb2	200517	200517	No	Intergenic region	
AG	CRAb2	200610	200610	No	Intergenic region	
GA	CRAb2	211490	211490	No (S)	TCG to TCA	putative outer membrane core complex of type IVb secretion system
GA	CRAb2	211795	211795	G to E	CGA to GAA	putative outer membrane core complex of type IVb secretion system
TA	CRAb2	217496	217496	L to I	TTA to ATA	Hypothetical protein
CA	CRAb2	220425	220425	P to Q	CCA to CAA	Hypothetical protein
TC	CRAb2	225355	225355	Y to H	TAT to CAT	DUF6685 domain-containing protein
AG	CRAb2	254368	254368	Stop to W	TAG to TGG	translesion error-prone DNA polymerase V autiproteolytic subunit
TC	CRAb2	255495	255495	M to T	ATG to ACG	DNA polymerase V subunit
TC	CRAb2	260925	260925	No (L)	CTT to CTC	ISAba126 family transposase
TC	CRAb2	261015	261015	No (T)	ACT to ACC	ISAba126 family transposase
TC	CRAb2	261018	261018	No (F)	TTT to TTC	ISAba126 family transposase
AC	CRAb2	261204	261204	No (P)	CCA to CCC	ISAba126 family transposase

**Deletions**						
ATGGTTTTAAG	CRAb1	121045	121055		Intergenic regions	repeat region
ACCACGCCAAGGAATCTAAAGCTGTTATCAGTCTGATAATGAAAAAACCA	CRAb1	200552	200601		Intergenic regions	
A	CRAb1	200546	200546		Intergenic regions	
**Insertions**						
ACCA	CRAb2	203730	203733		Intergenic regions	
**Transposons**						
tnp-IS30	CRAb1	171736	172746		Gene disruption	Hypothetical protein

Supplemental table 2. Complete annotation of the CRAb1 plasmid.

locus_tag	Name	product	Type	Gene start	Gene end	Length	Direction
	traJ-II RNA	traJ-II RNA regulatory	regulatory	116804	116904	101	reverse
LLILAP_18375		LPD1 domain-containing protein	CDS	173636	180862	7227	forward
LLILAP_18380		hypothetical protein	CDS	180872	181303	432	forward
LLILAP_18385		hypothetical protein	CDS	181576	182148	573	forward
LLILAP_18390		hypothetical protein	CDS	182102	182890	789	forward
LLILAP_18395		hypothetical protein	CDS	182899	183381	483	forward
LLILAP_18400		hypothetical protein	CDS	183479	185335	1857	forward
LLILAP_18405		hypothetical protein	CDS	185384	185785	402	reverse
LLILAP_18410		hypothetical protein	CDS	185835	186248	414	reverse
LLILAP_18415	dotA/traY	DotA/TraY family protein	Plasmid backbone	186515	189520	3006	forward
LLILAP_18420		hypothetical protein	CDS	189582	190076	495	reverse
LLILAP_18425		Peptidase family M23	Virulence related	190320	192332	2013	forward
LLILAP_18430		hypothetical protein	CDS	192355	192765	411	forward
LLILAP_18435		hypothetical protein	CDS	192780	193205	426	forward
LLILAP_18440		hypothetical protein	CDS	193258	193902	645	reverse
LLILAP_18445		ATP-dependent helicase	Plasmid backbone	193895	195451	1557	reverse
LLILAP_18450		hypothetical protein	CDS	195455	196012	558	reverse
LLILAP_18455		hypothetical protein	CDS	196012	196581	570	reverse
LLILAP_18460		hypothetical protein	CDS	196594	197268	675	reverse
LLILAP_18465		hypothetical protein	CDS	197281	198186	906	reverse
LLILAP_18470	icmP/dotM	Type IV secretion system protein IcmP/DotM	Plasmid backbone	198309	199838	1530	reverse
LLILAP_18475		NUBPL iron-transfer P-loop NTPase	Virulence related	201241	202077	837	forward
LLILAP_18480	parB	ParB domain-containing protein	Virulence related	202087	203148	1062	forward
LLILAP_18485		hypothetical protein	CDS	203194	204021	828	reverse
LLILAP_18490	stbA	StbA protein	Virulence related	204018	205124	1107	reverse
LLILAP_18495		hypothetical protein	CDS	205151	205420	270	reverse
LLILAP_18500		Type II/IV secretion system protein	Virulence related	205430	206725	1296	reverse
LLILAP_18505		Type IV secretion system, conjugal DNA-protein transfer	Plasmid backbone	206753	207562	810	reverse
LLILAP_18510	dotD/traH	DotD/TraH family lipoprotein	Plasmid backbone	207600	208046	447	reverse
LLILAP_18515		hypothetical protein	CDS	209131	210354	1224	forward
LLILAP_18520		hypothetical protein	CDS	210355	210642	288	forward
LLILAP_18525		Type-IV b secretion system, inner-membrane complex component	Plasmid backbone	210653	211336	684	forward
LLILAP_18530		Putative outer membrane core complex of type IVb secretion system	Plasmid backbone	211350	212297	948	forward
LLILAP_18535	trbI	DotG/IcmE/VirB10 family protein	Plasmid backbone	212319	213830	1512	forward
LLILAP_18540		hypothetical protein	CDS	213836	214717	882	forward
LLILAP_18545		hypothetical protein	CDS	214728	215303	576	forward
LLILAP_18550		hypothetical protein	CDS	215471	215884	414	forward
LLILAP_18555		HNH endonuclease	regulatory	215995	216882	888	forward
LLILAP_18560		hypothetical protein	CDS	216893	218095	1203	forward

LLILAP_18565	icmB/dotO	type IV secretion system protein IcmB/DotO	Plasmid backbone	218153	221335	3183	forward
LLILAP_18570	dotL	type IV secretion protein DotL	Plasmid backbone	221458	224250	2793	forward
LLILAP_18575		hypothetical protein	CDS	224885	225058	174	forward
LLILAP_18580		DUF6685 domain-containing protein	CDS	225145	226233	1089	forward
LLILAP_18585		hypothetical protein	CDS	226246	226752	507	forward
LLILAP_18590	int	Phage integrase family	Mobile genetic elements	227512	228177	666	forward
LLILAP_18595	tet(X3)	tetracycline-inactivating monooxygenase Tet(X3)	Antibiotic resistance	228367	229533	1167	forward
LLILAP_18600	spoIVCA	resolvase	Plasmid backbone	229682	230419	738	forward
LLILAP_18605		hypothetical protein	CDS	230416	230640	225	forward
LLILAP_18610	tnp-ISVsa3	IS91-like element ISVsa3 family transposase	Mobile genetic elements	230851	232344	1494	forward
LLILAP_18615		phosphoglucosamine mutase	Virulence related	232520	232822	303	reverse
LLILAP_18620	sul2	sulfonamide-resistant dihydropteroate synthase Sul2	Antibiotic resistance	232909	233757	849	reverse
LLILAP_18625	tnp-ISAba1	IS4 family ISAba1 transposase	Mobile genetic elements	233814	234383	570	forward
LLILAP_18630	tnp-IS4	IS4 family transposase	Mobile genetic elements	234766	234903	138	forward
LLILAP_18635		hypothetical protein	CDS	234900	235205	306	forward
LLILAP_18640		hypothetical protein	CDS	235303	235812	510	forward
LLILAP_18645		hypothetical protein	CDS	235952	236938	987	forward
LLILAP_18650		hypothetical protein	CDS	236994	237371	378	reverse
LLILAP_18655		hypothetical protein	CDS	237385	237744	360	reverse
LLILAP_18660		hypothetical protein	CDS	238277	239227	951	reverse
LLILAP_18665		hypothetical protein	CDS	239239	239709	471	reverse
LLILAP_18670		hypothetical protein	CDS	239774	240181	408	reverse
LLILAP_18675		hypothetical protein	CDS	240731	241120	390	forward
LLILAP_18680		hypothetical protein	CDS	241371	242819	1449	forward
LLILAP_18685		hypothetical protein	CDS	242897	243301	405	forward
LLILAP_18690		HlyD-D23 domain-containing protein	Antibiotic resistance	243678	244841	1164	forward
LLILAP_18695	adeB	multidrug efflux RND transporter permease subunit	Antibiotic resistance	244841	247942	3102	forward
LLILAP_18700		MFS transporter	Virulence related	248069	249583	1515	forward
LLILAP_18705	AdeS	two-component sensor histidine kinase AdeS	Antibiotic resistance	249687	250403	717	reverse
LLILAP_18710		hypothetical protein	CDS	250458	250748	291	reverse
LLILAP_18715	adeR	efflux system response regulator transcription factor AdeR	Antibiotic resistance	250792	251520	729	reverse
LLILAP_18720		hypothetical protein	CDS	251644	252537	894	reverse
LLILAP_18725	tnp-ISAlw15	IS5 family ISAlw15 transposase	Mobile genetic elements	252808	253212	405	forward
LLILAP_18730	tnp-IS5	IS5 family transposase	Mobile genetic elements	253415	253642	228	forward
LLILAP_18735		Translesion error-prone DNA polymerase V autoproteolytic subunit	Virulence related	253848	254369	522	forward
LLILAP_18740	umuC	DNA polymerase V subunit UmuC	Virulence related	254456	255694	1239	forward
LLILAP_18745		hypothetical protein	CDS	255829	256116	288	reverse
LLILAP_18750		hypothetical protein	CDS	256606	257034	429	reverse
LLILAP_18755		hypothetical protein	CDS	257057	258013	957	reverse
LLILAP_18760		hypothetical protein	CDS	258349	258774	426	reverse

LLILAP_18765		hypothetical protein	CDS	258813	258968	156	reverse
LLILAP_18770		hypothetical protein	CDS	259151	260299	1149	reverse
LLILAP_18775	tnp-ISAba125	IS30-like element ISAba125 family transposase	Mobile genetic elements	260833	261843	1011	forward
LLILAP_18780	blaNDM-1	subclass B1 metallo-beta-lactamase NDM-1	Antibiotic resistance	1	813	813	forward
LLILAP_18785		hypothetical protein	CDS	694	1245	552	reverse
LLILAP_18790	ble	Bleomycin resistance protein	Antibiotic resistance	2142	3023	882	forward
LLILAP_18795		hypothetical protein	CDS	2968	3258	291	forward
LLILAP_18800	groES	co-chaperone GroES	Virulence related	3395	3685	291	forward
LLILAP_18805	tnp-IS91	IS91 family transposase	Mobile genetic elements	5570	7099	1530	forward
LLILAP_18810		Acyltransferase family protein	Virulence related	7310	7594	285	reverse
LLILAP_18815	tnp-ISAba125	IS30-like element ISAba125 family transposase	Mobile genetic elements	7902	8912	1011	forward
LLILAP_18820		hypothetical protein	CDS	8847	9374	528	reverse
LLILAP_18825		hypothetical protein	CDS	9367	10239	873	reverse
LLILAP_18830		hypothetical protein	CDS	10252	10893	642	reverse
LLILAP_18835		hypothetical protein	CDS	11114	11644	531	reverse
LLILAP_18840		hypothetical protein	CDS	11845	12327	483	reverse
LLILAP_18845		DUF6573 domain-containing protein	CDS	12407	12841	435	reverse
LLILAP_18850		cold shock domain-containing protein	Virulence related	12992	13489	498	forward
LLILAP_18855		hypothetical protein	CDS	13559	13897	339	reverse
LLILAP_18860		phosphohydrolase	Virulence related	14055	15152	1098	reverse
LLILAP_18865		hypothetical protein	CDS	15329	15805	477	reverse
LLILAP_18870		hypothetical protein	CDS	15878	16192	315	reverse
LLILAP_18875		hypothetical protein	CDS	16205	17047	843	reverse
LLILAP_18880		hypothetical protein	CDS	17299	17829	531	reverse
LLILAP_18885		hypothetical protein	CDS	17873	18436	564	reverse
LLILAP_18890	fic/DOC	Fic/DOC family	Plasmid backbone	18581	19339	759	reverse
LLILAP_18895		hypothetical protein	CDS	19409	19621	213	reverse
LLILAP_18900		hypothetical protein	CDS	19819	20274	456	forward
LLILAP_18905		hypothetical protein	CDS	20377	20913	537	reverse
LLILAP_18910		hypothetical protein	CDS	21525	22019	495	reverse
LLILAP_18915		hypothetical protein	CDS	22113	22538	426	reverse
LLILAP_18920		hypothetical protein	CDS	22580	23473	894	reverse
LLILAP_18925		hypothetical protein	CDS	23473	23901	429	reverse
LLILAP_18930		hypothetical protein	CDS	24129	24953	825	reverse
LLILAP_18935		HNS domain-containing protein	CDS	25140	25466	327	forward
LLILAP_18940		hypothetical protein	CDS	25546	25815	270	reverse
LLILAP_18945		hypothetical protein	CDS	25815	26159	345	reverse
LLILAP_18950		hypothetical protein	CDS	26320	26508	189	forward
LLILAP_18955	tnp-IS4	IS4 family transposase	Mobile genetic elements	26867	27403	537	reverse
LLILAP_18960	tnp-IS4	IS4 family transposase	Mobile genetic elements	27400	27960	561	reverse
LLILAP_18965		hypothetical protein	CDS	28019	28219	201	forward
LLILAP_18970	gstA	glutathione S-transferase	Virulence related	28336	28953	618	forward

LLILAP_18975		hypothetical protein	CDS	29045	29398	354	forward
LLILAP_18980	yqaA	Inner membrane protein YqaA	Virulence related	29395	29823	429	forward
LLILAP_18985		MFS transporter	Virulence related	29859	31112	1254	reverse
LLILAP_18990	tnp-IS4	IS4 family transposase	Mobile genetic elements	31229	31789	561	forward
LLILAP_18995	tnp-IS4	IS4 family transposase	Mobile genetic elements	31786	32322	537	forward
LLILAP_19000		hypothetical protein	CDS	32317	33234	918	reverse
LLILAP_19005	cas6/cse3/casE	Type I-E CRISPR-associated protein Cas6/Cse3/CasE	Plasmid backbone	33311	34042	732	reverse
LLILAP_19010	mpr	zinc metalloproteinase Mpr protein	Virulence related	34335	35513	1179	forward
LLILAP_19015		hypothetical protein	CDS	35510	35878	369	forward
LLILAP_19020		helicase SNF2	Plasmid backbone	35878	37545	1668	forward
LLILAP_19025		hypothetical protein	CDS	37556	37672	117	forward
LLILAP_19030	tnp-IS3	IS3 family transposase	Mobile genetic elements	37831	38523	693	reverse
LLILAP_19035	tnp-ISAba14	IS3 family ISAba14 transposase	Mobile genetic elements	38658	38963	306	reverse
LLILAP_19040		hypothetical protein	CDS	39030	39518	489	reverse
LLILAP_19045	merA	mercury(II) reductase	Antibiotic resistance	39663	41348	1686	reverse
LLILAP_19050		hypothetical protein	CDS	41435	41845	411	forward
LLILAP_19055	merR	mercury resistance transcriptional regulator MerR	Antibiotic resistance	42248	42703	456	forward
LLILAP_19060	tnp-IS5	IS5 family transposase	Mobile genetic elements	42710	43135	426	reverse
LLILAP_19065		hypothetical protein	CDS	43324	43668	345	forward
LLILAP_19070		hypothetical protein	CDS	43730	43948	219	forward
LLILAP_19075		hypothetical protein	CDS	44667	45623	957	reverse
LLILAP_19080		hypothetical protein	CDS	45780	46430	651	forward
LLILAP_19085	traX	TraX protein	Plasmid backbone	46406	47197	792	forward
LLILAP_19090		hypothetical protein	CDS	47266	48012	747	reverse
LLILAP_19095		hypothetical protein	CDS	48130	48870	741	reverse
LLILAP_19100		hypothetical protein	CDS	48905	49474	570	reverse
LLILAP_19105		hypothetical protein	CDS	49525	49947	423	reverse
LLILAP_19110		hypothetical protein	CDS	50007	50813	807	forward
LLILAP_19115		SLT domain-containing protein	CDS	50815	51669	855	forward
LLILAP_19120		TNase-like domain-containing protein	CDS	51755	52261	507	forward
LLILAP_19125		hypothetical protein	CDS	52267	52575	309	forward
LLILAP_19130		site-specific DNA-methyltransferase	regulatory	52748	54283	1536	forward
LLILAP_19135		restriction endonuclease subunit R	Plasmid backbone	54299	56986	2688	forward
LLILAP_19140		hypothetical protein	CDS	57039	59882	2844	reverse
LLILAP_19145		DUF2807 domain-containing protein	CDS	60180	60842	663	forward
LLILAP_19150		virulence-associated e family protein	Virulence related	60987	62666	1680	reverse
LLILAP_19155		hypothetical protein	CDS	62683	63591	909	reverse
LLILAP_19160		hypothetical protein	CDS	63660	64913	1254	reverse
LLILAP_19165		hypothetical protein	CDS	65515	65748	234	forward
LLILAP_19170		hypothetical protein	CDS	65799	65951	153	reverse
LLILAP_19175		hypothetical protein	CDS	65951	66472	522	reverse

LLILAP_19180		type I DNA topoisomerase	Plasmid backbone	66828	69491	2664	forward
LLILAP_19185		hypothetical protein	CDS	69508	70506	999	forward
LLILAP_19190		hypothetical protein	CDS	70702	70935	234	forward
LLILAP_19195		zinc-ribbon-2 domain-containing protein	CDS	71016	72824	1809	reverse
LLILAP_19200		hypothetical protein	CDS	73026	73355	330	reverse
LLILAP_19205		H-NS histone family protein	CDS	73598	74059	462	reverse
LLILAP_19210		DUF2726 domain-containing protein	CDS	74171	74689	519	reverse
LLILAP_19215		hypothetical protein	CDS	74908	75597	690	forward
LLILAP_19220		hypothetical protein	CDS	75978	76361	384	forward
LLILAP_19225		NERD domain-containing protein	regulatory	76364	77200	837	forward
LLILAP_19230		hypothetical protein	CDS	77313	78350	1038	reverse
LLILAP_19235		hypothetical protein	CDS	78462	80378	1917	forward
LLILAP_19240		hypothetical protein	CDS	80498	81343	846	forward
LLILAP_19245		hypothetical protein	CDS	81459	82031	573	forward
LLILAP_19250	int	Phage integrase family	Mobile genetic elements	82058	82990	933	forward
LLILAP_19255		hypothetical protein	CDS	83156	83818	663	forward
LLILAP_19260	tn3	Tn3 family transposase	Mobile genetic elements	83904	86330	2427	reverse
LLILAP_19265	aph(3')-Ia	aminoglycoside O-phosphotransferase APH(3')-Ia	Antibiotic resistance	86932	87795	864	reverse
LLILAP_19270	ant(2'')-Ia	aminoglycoside nucleotidyltransferase ANT(2'')-Ia	Antibiotic resistance	87846	88379	534	reverse
LLILAP_19275	tnp-IS26	IS6-like element IS26 family transposase	Mobile genetic elements	88516	89220	705	forward
LLILAP_19280	tn5393	Transposase of Tn5393-like transposon, N-terminal	Mobile genetic elements	89254	90330	1077	reverse
LLILAP_19285	spoIVCA	transposon DNA-invertase	Virulence related	90456	91070	615	forward
LLILAP_19290	tnp-ISAba14	IS3 family ISAba14 transposase	Mobile genetic elements	91189	91494	306	forward
LLILAP_19295	tnp-IS3	IS3 family transposase	Mobile genetic elements	91629	92321	693	forward
LLILAP_19300	aph(3'')-Ib	aminoglycoside O-phosphotransferase APH(3'')-Ib	Antibiotic resistance	92421	93224	804	forward
LLILAP_19305	aph(6)-Id	aminoglycoside O-phosphotransferase APH(6)-Id	Antibiotic resistance	93224	94060	837	forward
LLILAP_19310		plasmid replication DNA-binding protein	Plasmid backbone	94217	94669	453	forward
LLILAP_19315		hypothetical protein	CDS	94691	94834	144	forward
LLILAP_19320		hypothetical protein	CDS	95033	95491	459	reverse
LLILAP_19325		hypothetical protein	CDS	95491	96225	735	reverse
LLILAP_19330	tnp-IS30	IS30 family transposase	Mobile genetic elements	96382	97392	1011	forward
LLILAP_19335	tnp-IS1	IS1 family transposase	Mobile genetic elements	97421	97867	447	reverse
LLILAP_19340	blaOXA-58	OXA-58 family carbapenem-hydrolyzing class D beta-lactamase	Antibiotic resistance	98017	98790	774	forward
LLILAP_19345	tnp-IS1	IS1 family transposase	Mobile genetic elements	98881	99318	438	forward
LLILAP_19350	araC	AraC family transcriptional regulator	regulatory	99761	100588	828	forward
LLILAP_19355	lysE	LysE family translocator	regulatory	100638	101243	606	forward
LLILAP_19360	relE/parE	addiction module killer protein	Plasmid backbone	101340	101636	297	forward
LLILAP_19365		putative addiction module antidote protein	regulatory	101638	101928	291	forward

LLILAP_19370	tnp-ISNCY	ISNCY family transposase	Mobile genetic elements	102031	103365	1335	reverse
LLILAP_19375		hypothetical protein	CDS	103508	103990	483	forward
LLILAP_19380	relE/parE	Type II toxin-antitoxin system RelE/ParE family toxin	Plasmid backbone	104106	104426	321	forward
LLILAP_19385	nadS	NadS family protein	regulatory	104419	104691	273	forward
LLILAP_19390	tnp-ISNCY	ISNCY family transposase	Mobile genetic elements	104787	106121	1335	reverse
LLILAP_19395	msr(E)	ABC-F type ribosomal protection protein Msr(E)	Antibiotic resistance	106670	108145	1476	forward
LLILAP_19400	mph(E)	Mph(E) family macrolide 2'-phosphotransferase	Antibiotic resistance	108201	109085	885	forward
LLILAP_19405		resolvase	Virulence related	109275	109886	612	reverse
LLILAP_19410	lrp	DNA-binding transcriptional regulator, Lrp family	Virulence related	110085	110567	483	reverse
LLILAP_19415	lysE	Lysine transporter LysE	regulatory	110698	111333	636	forward
LLILAP_19420		hypothetical protein	CDS	111791	112105	315	reverse
LLILAP_19425		Iron ABC transporter permease	Virulence related	112412	114040	1629	reverse
LLILAP_19430		iron ABC transporter substrate-binding protein	Virulence related	114124	115134	1011	reverse
LLILAP_19435	fbpC 2	Fe(3+) ions import ATP-binding protein FbpC 2	Virulence related	115157	116281	1125	reverse
LLILAP_19440	lysR	LysR family transcriptional regulator	regulatory	116400	116768	369	reverse
LLILAP_19445		hypothetical protein	CDS	117074	117409	336	forward
LLILAP_19450	tnp-ISAlw25	IS1 family ISAlw25 transposase	Mobile genetic elements	117809	118510	702	reverse
LLILAP_19455		hypothetical protein	CDS	118834	119079	246	forward
LLILAP_19460		hypothetical protein	CDS	119155	119583	429	reverse
LLILAP_19465		hypothetical protein	CDS	119773	120306	534	forward
LLILAP_19470		N-acetylmuramoyl-L-alanine amidase	regulatory	120345	120986	642	reverse
LLILAP_19475		hypothetical protein	CDS	121157	121909	753	forward
LLILAP_19480		hypothetical protein	CDS	122297	122542	246	reverse
LLILAP_19485	pcfJ	PcfJ-like protein	CDS	122734	124404	1671	reverse
LLILAP_19490		LPD1 domain-containing protein	CDS	124401	126041	1641	reverse
LLILAP_19495		hypothetical protein	CDS	126060	126443	384	reverse
LLILAP_19500		hypothetical protein	CDS	126450	127217	768	reverse
LLILAP_19505		Putative lipoprotein	Virulence related	127219	128541	1323	reverse
LLILAP_19510		hypothetical protein	CDS	128632	129150	519	reverse
LLILAP_19515		hypothetical protein	CDS	129225	129578	354	forward
LLILAP_19520		hypothetical protein	CDS	129678	130424	747	reverse
LLILAP_19525		Putative lipoprotein	Virulence related	130485	130847	363	forward
LLILAP_19530		hypothetical protein	CDS	130997	131890	894	reverse
LLILAP_19535	loiP	Metalloprotease LoiP	Virulence related	131906	132685	780	reverse
LLILAP_19540		hypothetical protein	CDS	132678	133166	489	reverse
LLILAP_19545		hypothetical protein	CDS	133176	133685	510	reverse
LLILAP_19550		hypothetical protein	CDS	133691	134113	423	reverse
LLILAP_19555		hypothetical protein	CDS	134278	134931	654	forward
LLILAP_19560		hypothetical protein	CDS	134997	135509	513	reverse
LLILAP_19565		hypothetical protein	CDS	135517	136647	1131	reverse
LLILAP_19570		hypothetical protein	CDS	137333	138202	870	reverse

LLILAP_19575		Transporter	Virulence related	138304	138903	600	reverse
LLILAP_19580		hypothetical protein	CDS	138896	140686	1791	reverse
LLILAP_19585		hypothetical protein	CDS	140854	141330	477	reverse
LLILAP_19590		hypothetical protein	CDS	143186	144820	1635	forward
LLILAP_19595	res	Type III restriction enzyme, res subunit	Plasmid backbone	144852	151484	6633	forward
LLILAP_19600		hypothetical protein	CDS	151614	151763	150	forward
LLILAP_19605		hypothetical protein	CDS	151797	156230	4434	forward
LLILAP_19610		hypothetical protein	CDS	156233	157126	894	forward
LLILAP_19615		hypothetical protein	CDS	157195	157623	429	forward
LLILAP_19620		hypothetical protein	CDS	157627	158433	807	forward
LLILAP_19625		hypothetical protein	CDS	158459	159421	963	forward
LLILAP_19630		hypothetical protein	CDS	159434	160270	837	forward
LLILAP_19635		hypothetical protein	CDS	160276	161466	1191	forward
LLILAP_19640		hypothetical protein	CDS	161479	162720	1242	forward
LLILAP_19645		LPD1 domain-containing protein	CDS	162930	170216	7287	forward
LLILAP_19650		hypothetical protein	CDS	170300	170959	660	forward
LLILAP_19655		hypothetical protein	CDS	171132	171683	552	forward
LLILAP_19660	tnp-IS30	IS30 family transposase	Mobile genetic elements	171736	172746	1011	forward
LLILAP_19665		hypothetical protein	CDS	172795	173394	600	forward
	gi2	Genomic island 2	Mobile genetic elements	82058	94060	12003	
	gi3	Genomic island 3	Mobile genetic elements	227510	234383	6874	
	gi1	Genomic island 1	Mobile genetic elements	694	7099	6406	
